# Ekbom Syndrome: what do we know about it? A case report

**DOI:** 10.1192/j.eurpsy.2025.2241

**Published:** 2025-08-26

**Authors:** C. Munaiz Cossío, I. M. Peso Navarro, N. M. Casado Espada, M. Ligero Argudo, R. K. Gonzalez Bolaños, C. Payo Rodriguez, J. I. de la Iglesia Larrad, P. Andres Olivera

**Affiliations:** 1 Psychiatry, Complejo Asistencial Sanitario de Salamanca, Salamanca, Spain

## Abstract

**Introduction:**

Ekbom Syndrome or delusional parasitosis is a, usually monosymptomatic, somatic type of delusional disorder in which the patients are convinced they are being infested with animal parasites while no objective evidence exists to support this belief (Mumcuoglu KY, Leibovici V, Reuveni I, Bonne O. Isr Med Assoc J. 2018 Jul;20(7):456-460). It has also been described as a hypochondriacal psychosis that causes great suffering for the patient and those around them. (Campbell EH, Elston DM, Hawthorne JD, Beckert DR. J Am Acad Dermatol. 2019 May;80(5):1428-1434.)

**Objectives:**

To study this syndrome in depth and learn more about these patients.

**Methods:**

The Pubmed database was used to collect the available information about Ekbom syndrome since 2006. Using the search term “delusional parasitosis”. We also present the following clinical case:

A 66-year-old woman, with no history of mental health or substance abuse, came to the dermatology department for multiple pruritic wounds all over her body. She reports that when she scratches herself there is a “small ball that crackles, like a living thing”, she has tried to bring samples but has not been able to collect them, according to her. She is convinced that she is infested by parasites. In addition, in the last few weeks she has suffered significant hair loss. On physical examination she presents multiple scabby plaques on the thorax, back, face and scalp. On the scalp she presents frontal alopecia and madarosis.

The patient was referred to Psychiatry.

On psychopathological examination the patient presented moderate ideational and somatic anxiety, reactive to hair loss and feeling of infestation, also admits difficulty in falling asleep. No affective symptoms were observed or described, nor were there any other accompanying psychotic symptoms (self-referentiality, delusions of harm, persecution, etc.).

**Results:**

We started treatment with Olanzapine 5mg before going to bed, plus a rescue tablet if needed due to anxiety.

In the following reviews, the delirious clinic persists, although a notable reduction of the anxiety and improvement of the night rest is observed.

**Conclusions:**

When it comes to treatment, one of the main difficulties we find is that the patient accepts to be evaluated by psychiatry, so it is important to establish a good therapeutic relationship with the patient, since this will determine the patient’s therapeutic adherence to the treatment.

Treatment usually consists of antipuriginous and antipsychotic agents. In our case we selected Olanzapine for its sedative effect, with which we observed a significant reduction of symptoms. However, the available literature usually recommends starting treatment with risperidone. Even so, there is little evidence on its efficacy in Eckbom’s Syndrome.

Treatment studies are scarce and the evolution varies from one case to another.

**Disclosure of Interest:**

None Declared

